# Editorial: Steroid hormone receptors in cardiometabolic disease

**DOI:** 10.3389/fendo.2023.1251897

**Published:** 2023-07-19

**Authors:** Julie Goodwin, Alex Odermatt, Ruth Morgan, Mark Nixon

**Affiliations:** ^1^ Pediatrics, Yale University, New Haven, CT, United States; ^2^ Department of Pharmaceutical Sciences, University of Basel, Basel, Switzerland; ^3^ Animal and Veterinary Sciences, Scotland's Rural College, Edinburgh, United Kingdom; ^4^ Centre for Cardiovascular Science, University of Edinburgh, Edinburgh, United Kingdom

**Keywords:** steroid hormone receptor, cardiometabolic health, cardiovascular disease risk, steroid hormones and receptors, steroid hormones

Cardiovascular disease (CVD) remains the leading cause of death worldwide, with over 17 million deaths annually. Although the incidence of CVD has been declining, this has slowed in recent years, arguably because of the substantial increase in obesity which in turn amplifies cardiovascular risk factors including metabolic dysfunction (hyperglycemia, dyslipidemia, hypertension) and chronic kidney disease (1). There is a critical unmet need for therapies which target metabolic dysfunction and thereby ameliorate its consequences for cardiovascular disease risk.

Steroid hormones are crucial regulators of physiological processes including stress regulation, immune function, sexual development, fuel metabolism and cellular growth and differentiation. Mainly produced by the adrenal glands and gonads, they are grouped into five classes: androgens, estrogens, glucocorticoids, mineralocorticoids and progestogens. Since the discovery of adrenal insufficiency by Thomas Addison in 1855 (2), numerous studies have characterized the influence of different steroid hormones, including cortisol and testosterone, on cardiovascular disease and its associated risk factors (3, 4). Defining the molecular mechanisms underlying the cardiometabolic actions of these steroid hormones remains a central challenge in the field of endocrinology. To this day, the ‘two-step’ model for steroid action proposed by Jensen and Gorski in 1968 (5) is pivotal in understanding steroid hormone action, depicting binding of the hormone to a specific high-affinity receptor within target cells, followed by the activation of hormone-receptor complex and the resulting induction of hormone-responsive genes. However, our knowledge of steroid hormone receptor (SHR) action continues to advance as we seek to identify the role of SHRs in metabolic and cardiovascular disease. In this Research Topic of *Frontiers in Endocrinology*, we discuss the crucial role of steroid hormone receptors in cardiometabolic endocrinology.

First, Kober et al. explore the differences in glucocorticoid receptor (GR, NR3C1) and mineralocorticoid receptor (MR, NR3C2) expression in functioning vs. clinically silent neuroendocrine corticotroph tumors. Interestingly, they demonstrate that GR mRNA expression is higher in non-functioning tumors compared to those that result in clinically significant Cushing’s disease. Further, higher MR mRNA expression is observed in patients in remission from Cushing’s disease after surgery, suggesting that higher steroid receptor expression correlates with more favorable clinical outcomes. Follow-on studies on the regulation of GR and MR activity at the post-translational level and through co-regulatory proteins are warranted.

Next, Frigerio et al. examine glucocorticoid replacement dosing regimens in subjects with adrenal insufficiency (AI), a patient group in which steroid therapy significantly elevates the risk of cardiovascular disease. They present data supporting a potential rationale for switching from standard cortisone acetate (CA) or immediate-release hydrocortisone therapy to modified release hydrocortisone (MRH) therapy, citing improved blood pressure control and smoothing of the clinical symptoms of exogenous steroid therapy. This study highlights the balancing act that is often required when replacement steroids are necessary, taking into account not only potential differences in pharmacokinetics, but the altered steroid-receptor affinities and the subsequent consequences for tissue-specific downstream activation. This fine balance, which is inherent in endogenous steroid signaling, is further emphasized in work by Koorneef et al., in which the authors emphasize that endogenous glucocorticoids exert action not only through GR, but also through MR. This concept is explored in depth in a mouse study which demonstrates the interplay between both GR activation by synthetic dexamethasone and MR activation with endogenous corticosterone, and ultimately suggests that MR contributes to observed steroid effects, especially in the setting of synthetic glucocorticoid use. This may be especially relevant in tissues expressing GR and MR in the presence of 11β-HSD1 such as the adipose.

The study by Zhang et al. focuses on the importance of adrenal androgens. In a large cross-sectional study they evaluate associations of dehydroepiandrosterone (DHEA) and dehydroepiandrosterone sulfate (DHEAS), important precursors to the sex steroids testosterone and estradiol, with coronary heart disease and stroke in older men and women who also had type II diabetes mellitus. The authors demonstrate that both DHEA and DHEAS levels are inversely correlated with the risk of coronary heart disease in men, but no relationship was found between these levels and coronary heart disease in women. Further, the risk of stroke in either gender is not associated with these hormone levels. This study raises important considerations about the sex-specific differences in cardiovascular risk throughout the lifespan, many of which remain poorly understood and warrants further research.

Finally, Verma et al. round out the collection with a rigorous computational study about the ligand-binding domain (LBD) of the thyroid hormone-like (THR-like) receptor family. The authors aim to predict key residues responsible for the functional specificity of THR-like receptors, which are critical modulators of metabolic homeostasis. This study leverages state-of-the-art computational tools to perform predictive modeling of fold-specific and function-specific residues in THR-like LBDs which could ultimately be used to rationally develop novel therapeutics or assist in the basic pathophysiologic understanding of disease. It will be interesting to attempt to experimentally validate the predictions from computational methods in follow-on studies.

## Conclusions

Steroid hormone receptors are ubiquitously expressed, yet precisely controlled, gateways to many homeostatic physiologic processes, and cardiometabolic processes in particular. In some cases, the understanding of SHR-ligand interactions remains incomplete even under basal conditions, but what has become readily apparent over the last five decades of study in steroid hormone biology is that perturbations in SHRs are a major driver of human cardiovascular disease. As biological tools continue to become more sophisticated, we anticipate continued strides in our mechanistic understanding of the myriad roles of SHRs in human health, which will ultimately help to improve therapeutic applications.

## Author contributions

JG wrote the manuscript, AO, RM, and MN reviewed and edited the manuscript. All authors contributed to the article and approved the submitted version.
